# Air Jet Impingement Baking of Bread: Heat–Mass Transfer Dynamics and Process Optimization

**DOI:** 10.1111/1750-3841.71456

**Published:** 2026-09-10

**Authors:** Özgül Altay, Özge Filiz, Gönül Çavuşoğlu, Safiye Nur Dirim, Utku Şentürk, Figen Kaymak‐Ertekin

**Affiliations:** ^1^ Faculty of Engineering, Department of Food Engineering Ege University İzmir Türkiye; ^2^ Arçelik A.Ş İstanbul Turkey; ^3^ Faculty of Engineering, Department of Mechanical Engineering Ege University İzmir Türkiye

**Keywords:** air jet impingement baking, bread baking, energy consumption, impingement heat transfer, process optimization

## Abstract

Air jet impingement baking is a high‐intensity convective heating technology that enhances heat transfer efficiency by reducing thermal boundary layer resistance at the product surface. This study investigated the application of air jet impingement to bread baking, a volumetric food system with complex heat and mass transfer dynamics. Baking experiments were conducted at varying oven temperatures (165–195°C), air velocities (12–15 m/s), baking times (15–35 min), and tray positions using a central composite rotatable design. Product quality and process efficiency were evaluated through moisture content, height change, bulk density, bread volume, crust thickness, hardness, color parameters (a*, BI), springiness, hydroxymethylfurfural (HMF) formation, and energy consumption. Results indicated that temperature and baking time were the primary factors influencing structural development, browning, and HMF formation, whereas air velocity had a limited direct effect on most quality attributes. The intensified convective heat transfer in the impingement system accelerated surface heating and crust formation, achieving comparable or improved bread volume and texture at shorter baking times relative to conventional convection baking. Optimization analysis identified 195°C, 13.32 m/s air velocity, 35 min baking time, and lower tray position as the optimum conditions, providing balanced crust formation, desirable textural properties, and reduced energy consumption. Overall, air jet impingement baking demonstrates potential as an efficient alternative to conventional oven baking, improving heat transfer performance while maintaining product quality and minimizing formation of heat‐induced processing contaminants.

## 1 Introduction

Oven baking is a widely applied processing method used in the preparation of food products, encompassing both traditional practices and emerging technologies. During oven baking, heat is transferred to the food through conduction, convection, and radiation. Simultaneously, mass, energy, and momentum transfer processes occur, resulting in complex physical and chemical transformations within the food matrix (Bahram 1950).

During thermal processing, structural and compositional changes occur both at the surface and within the interior of the product. Moisture migrates from the core toward the surface due to temperature and vapor pressure gradients (Walker and Sparman 1989; İşleroğlu and Kaymak‐Ertekin 2016; Yoganarasimhaswamy et al. 2025). Achieving the target temperature at the geometric center (cold spot) is essential for uniform baking; however, heating generally occurs more rapidly at the surface than in the core, particularly in conventional ovens. This results in a non‐uniform temperature distribution within the product (Marston and Wannon 1976; Purlis 2023). Prolonged exposure to high temperatures may therefore lead to excessive surface dehydration, intensified browning reactions, and the formation of thermal processing‐induced compounds such as hydroxymethylfurfural (HMF), acrylamide, and heterocyclic amines (HCAs) (İşleroğlu et al. 2012, 2014).

HMF is a well‐known product of the Maillard reaction, a non‐enzymatic browning mechanism influenced by factors such as pH, temperature, water activity, and reactant composition (Mlotkiewicz 1998; Tressl and Rewicki 1999; Hidalgo and Zamora 2000; Zhang et al. 2025). Although certain Maillard reaction products may exhibit antioxidant properties, the formation of compounds such as HMF and acrylamide is generally undesirable due to their reported cytotoxic and carcinogenic potential (Borrelli et al. 2003; Lindermeier et al. 2002; Hosry et al. 2025). Acrylamide, first identified as a food safety concern in 2002, has been shown to be strongly influenced by processing parameters, including temperature, time, moisture content, and the characteristics of the food matrix (Sakin‐Yılmazer et al. 2013; Liu et al. 2025; Barutçu et al. 2009). Previous studies have also demonstrated that oven configuration can significantly affect acrylamide formation during baking (Sakin‐Yılmazer et al. 2013; Ahmad et al., 2022; Nguyen et al., 2022). Consequently, modern oven design has increasingly focused on improving heat transfer efficiency and reducing processing time to enhance both product quality and food safety.

Impinging air jet heat transfer is a high‐intensity convective heating technique widely used in heating and drying applications due to its high heat transfer coefficients. Unlike conventional ovens, where the formation of a thermal boundary layer limits heat transfer, high‐velocity air jets reduce the thickness of this boundary layer and deliver thermal energy directly to the product surface (Anderson 2004). This mechanism accelerates crust formation and color development, shortens processing time, and promotes more uniform heating, offering potential advantages for bakery applications. Although accelerated crust formation under high‐intensity convective heating may lead to the development of a low‐moisture surface layer that can partially restrict subsequent heat and moisture transfer toward the core, the substantially shorter processing times achieved in air‐jet impingement systems limit excessive crust drying and help mitigate this effect (Olsson et al. 2005).

Previous studies on air‐jet impingement baking have primarily focused on thin and low‐volume bakery products such as cakes (Li and Walker 1996; Xue and Walker 2003), par‐baked baguettes (Olsson et al. 2005), and flatbreads (Banooni et al. 2008a, 2009). Consistent with recent reviews, air‐jet impingement research has largely emphasized heat and mass transfer characteristics, flow behavior, heat transfer coefficients, equipment design, and process performance (Sarkar and Singh 2004; Altay et al. 2023), while comparatively fewer studies have investigated product quality in complex bakery systems (Altay et al. 2023). Furthermore, the available bread studies have mainly focused on thin or flat bread systems, whereas the application of air‐jet impingement baking to volumetric loaf bread remains largely unexplored. Consequently, the effects of air‐jet impingement on heat and mass transfer behavior, quality development, and contaminant formation in volumetric bread systems are still not well understood. (Banooni et al. 2008a, 2008b). In addition, no previous study has comprehensively integrated process optimization with technological quality attributes, energy consumption, and heat‐induced contaminant formation within a single experimental framework, limiting the practical evaluation of air‐jet impingement baking for industrial bread production. Accordingly, the present study was designed to address these specific knowledge gaps by providing the first comprehensive evaluation of air‐jet impingement baking for volumetric loaf bread through the integration of heat and mass transfer behavior, process optimization, product quality, energy consumption, and heat‐induced contaminant formation within a single experimental framework. Specifically, the objectives were: (i) to characterize heat and mass transfer behavior during air‐jet impingement baking by analyzing temperature profiles, moisture loss, and structural changes together with the resulting bread quality attributes; (ii) to optimize the main process variables (oven temperature, air velocity, baking time, and tray position) using Response Surface Methodology based on multiple quality responses; and (iii) to comparatively evaluate optimized air‐jet impingement and conventional baking in terms of product quality, energy consumption, and the formation of heat‐induced contaminants (HMF and acrylamide).

## 2 Materials and Methods

### 2.1 Materials

The bread mixes used for bread production were supplied by Pak Gıda A.Ş. The bread formulation consisted of 45% bread wheat flour, 45% commercial flour mix, 5% whole rye flour, 5% dark bran, and 0.02% ascorbic acid. The dough formulation comprised 301 g of the flour blend, 190 mL of water, 4.5 g of yeast, and 4.5 g of salt. Dough ingredients were mixed for 5 min until a homogeneous structure was obtained. The dough was then subjected to a two‐stage proofing process consisting of 30 min of bulk fermentation in the mixing tank followed by 30 min of final proofing in the baking molds prior to baking. To maintain a constant tray load during each baking experiment, approximately 500 g of dough was prepared for each batch. The dough was placed in a rectangular prism baking tray (8 × 27 × 7 cm) and filled to a height of 5 ± 0.2 cm. The initial dough volume was approximately 1080 cm^3^.

### 2.2 Air Jet Impingement Oven

**FIGURE 1:**
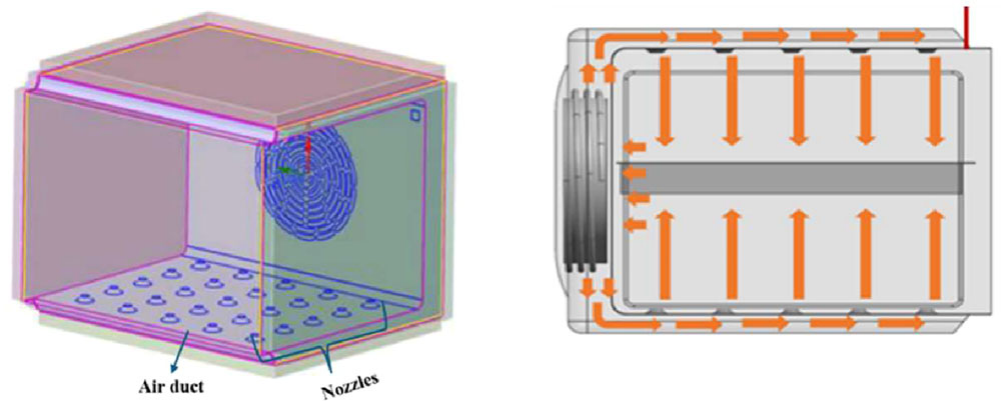
Schematic representation of the air jet impingement oven configuration.

Conventional baking experiments were carried out using a household‐type built‐in oven (Arçelik/Beko, model 13300XM) operated in turbo (forced convection) mode. The oven is equipped with a rear‐mounted radial fan–motor assembly, a turbo heating element, and a cast‐plate fan shield designed to facilitate hot air circulation within the baking chamber. Air is drawn toward the fan through the central opening of the shield and redistributed into the oven cavity through surrounding outlet perforations. A detailed description of the oven structure and airflow characteristics has been reported previously by Selçuk et al. (2026). The oven operates at a maximum temperature of 280°C and provides an average airflow rate of approximately 25 L/s^−^^1^. The internal baking chamber dimensions of both the conventional oven and the air jet impingement oven prototype were identical (60 × 60 cm), allowing direct comparison of the baking systems under comparable geometric conditions. Air velocity measurements indicated values of approximately 4.5 m s^−^^1^ at the fan outlet, 1–2 m s^−^^1^ at the baking tray positioned on the middle rack, and 1–1.5 m s^−^^1^ in the near‐surface region of the product (10–15 mm above the surface). To enable a direct comparison under similar structural conditions, an air jet impingement oven prototype was developed based on the same household oven model. In addition to the conventional configuration, the prototype was modified by integrating 50 air jet nozzles, uniformly distributed between the upper (25) and lower (25) sections of the oven cavity, to enhance convective heat transfer. A schematic representation of the air jet impingement oven is shown in Figure 1. The impingement oven is equipped with a 2500 W turbo heating element and operates at a maximum temperature of 280°C, providing an airflow rate of approximately 74 L/s^−^^1^. Under these operating conditions, the air velocity reaches a maximum of approximately 15 m s^−^^1^ at the nozzle exit and decreases to 4–4.5 m s^−^^1^ in the near‐surface region of the product (10–15 mm above the surface) when the fan operates at its maximum speed of 2500 rpm.

### 2.3 Baking Method in Air Jet Impingement Oven and Optimization of Baking Process

Baking experiments in the air jet impingement oven were conducted under the following operating conditions: baking trays were positioned either on the middle or lower rack; oven temperatures were set at 165, 180, and 195°C; air velocities were adjusted to 12 and 15 m s^−^^1^; and baking times were varied at 15, 25, and 35 min. The experimental ranges of oven temperature, air velocity, baking time, and the selected tray positions were established based on preliminary baking trials to ensure appropriate baking conditions and measurable differences in product quality. For comparison, baking trials were also performed in a conventional oven operating in turbo mode (air velocity ≈ 4.5 m/s^−^^1^) at 200°C for 45 min (manufacturer's recommended baking program). Turbo mode was selected because it is the manufacturer‐recommended and commonly used setting for bread baking in household ovens. In addition, this mode was chosen to enable a meaningful comparison with the air‐jet impingement prototype, as both systems operate on the principle of forced convective heat transfer. In all experiments, samples were placed on the middle rack, and each baking condition was duplicated.

Bread baking experiments were conducted according to predefined experimental conditions comprising 18 experimental runs generated from combinations of oven temperature, air velocity, baking time, and tray position. Oven temperature (A), air velocity (B), baking time (C), and tray position (D) were considered as independent variables for the optimization analysis. Tray position was incorporated into the regression model using binary coding (0 = middle rack, 1 = lower rack) to evaluate its linear effect. Since this factor had only two levels, no quadratic effect was estimated for tray position. The response variables included crust thickness, bread volume, and hardness. The experimental data obtained from these baking trials were subsequently analyzed and optimized using Response Surface Methodology (RSM) implemented in Design‐Expert software. Multiple regression analysis was used to fit the data to the second‐order polynomial model given in Equation (1). The regression coefficients were estimated using least‐squares regression based on coded variables. Model significance was evaluated using analysis of variance (ANOVA), while model adequacy was assessed using the coefficient of determination (*R* ^2^), adjusted *R* ^2^, predicted *R* ^2^, adequate precision, and lack‐of‐fit tests.

(1) $$Y\;=\beta_{0}+\sum_{i\;=1}^{k}\beta_{i}x_{i}+\sum_{i\;=1}^{k}\beta_{\mathrm{ii}}x_{i}^{2}+\sum_{i\;=1}^{k-1}\sum_{j\;=\;i+1}^{k}\beta_{\mathrm{ij}}x_{\mathrm{ij}}\;\left(k\;=1,2,3\right)$$

In this model, Y represents the predicted response, x denotes the independent variables, β_0_ is the intercept term, β_i_ represents the linear coefficients, β_i__i_ corresponds to the quadratic coefficients, and β_i__j_ denotes the interaction coefficients. The independent variables were mathematically modeled to determine both their individual and interaction effects on the response variables.

Finally, five additional baking experiments were conducted under the identified optimum conditions to validate the predicted results. All response variable analyses were repeated using these validation samples. The obtained data were subsequently analyzed using IBM SPSS Statistics (IBM Corp., Chicago, IL, USA) through paired‐sample t‐tests, confirming the adequacy of the predicted optimum conditions (*p* > 0.05).

### 2.4 Analysis

To ensure representative sampling, composite samples were prepared by homogenizing subsamples collected from the crust, crumb, and bottom sections of each bread sample. All physical and chemical analyses were performed in triplicate, except for texture measurements. For texture analysis, ten replicate measurements were obtained for each sample. Energy consumption was recorded during each baking experiment.

#### 2.4.1 Temperature Profile

During all baking experiments, temperature profiles were recorded using a temperature data logger (Hanna Instruments, HI 98804) equipped with J‐type thermocouples (1 mm diameter). The thermocouples were positioned at the upper surface, geometric center, and lower surface of the bread samples, and temperature changes were continuously monitored throughout the baking process. The thermocouple used for surface temperature measurements was fixed directly to the dough surface using a hook‐shaped configuration, allowing the sensor to move in accordance with dough expansion during baking. Oven temperature was measured using a separate thermocouple positioned at a fixed location within the oven cavity and monitored throughout the baking process.

#### 2.4.2 Moisture Content (%)

Moisture content of the bread samples was determined according to AOAC (1990). Approximately 3–4 g of sample was dried in an oven at 105°C until constant weight was achieved. Moisture content (%) was calculated based on weight loss during drying.

#### 2.4.3 Height Change (%) and Crust Thickness (Mm)

The height change and crust thickness of the bread samples were determined using a digital caliper by measuring the relevant dimensions before and after baking. Height change was calculated at the highest point of the baked bread samples.

#### 2.4.4 Bulk Density (g/cm^−3^) and Bread Volume (cm^3^)

Bread volume was determined using the rapeseed displacement method (AACC International Method 10‐05.01, 2010). Bread samples were placed in a graduated container filled with rapeseeds of known mass and volume. The volume of displaced seeds was measured and used to calculate the volume of the bread samples (cm^3^). Bulk density was calculated as the mass‐to‐volume ratio of the bread sample using the measured volume.

#### 2.4.5 Texture Profile Analysis (TPA)

Texture properties of the bread samples were determined using a texture analyzer (TA‐XT2, Stable Micro Systems, Haslemere, UK) equipped with a cylindrical probe (P/36R). Bread slices with dimensions of approximately 65 mm x 6 mm x 20 mm (length x width x thickness) were used for texture profile analysis (TPA). The pre‐test, test, and post‐test speeds were set to 2.0 mm s^−^^1^, with a test distance of 5 mm and a trigger force of 0.049 N.

Hardness was defined as the maximum force recorded during the first compression cycle of the force–distance curve. Springiness was calculated as the ratio of the recovery distance between the first and second compression cycles obtained from the TPA curve. Each measurement was performed in ten replicates, and mean values were reported (Liu et al. 2024).

#### 2.4.6 Color

L* (lightness), a* (redness), and b* (yellowness) values of the bread samples were measured using a Minolta CR‐400 colorimeter (Konica Minolta, Japan), and results were expressed according to the CIE Lab* color system. The browning index (BI) of the samples was calculated using Equation (2) (Dadalı et al. 2007) :

(2) $$\mathrm{BI}=\frac{100\times \left(\frac{a^{*}+1.75\times L^{*}}{5.645\times L^{*}+a-3.012\times b^{*}}-0.31\right)}{0.17}$$

Five measurements were performed for each sample, and the mean values were reported.

#### 2.4.7 Hydroxymethylfurfural (HMF) Analysis

The hydroxymethylfurfural (HMF) content of the baked bread samples was determined using the Winkler colorimetric method described by Bogdanov et al. (2002). Briefly, 20 g of the homogenized bread sample was diluted to 100 mL with distilled water and filtered through filter paper. From the filtrate, 2 mL aliquots were transferred into two test tubes (blank and sample). To each tube, 5 mL of p‐toluidine solution was added, followed by 1 mL of distilled water and 1 mL of barbituric acid solution. The mixtures were homogenized using a vortex mixer. Absorbance was measured at 550 nm using a UV–Vis spectrophotometer (Shimadzu UV‐1900) with quartz cuvettes. HMF content was calculated based on absorbance values and expressed as mg kg^−^^1^ product.

#### 2.4.8 Acrylamide Analysis

Acrylamide content of bread samples produced under optimum baking conditions was determined at Radix Analysis Laboratories using liquid chromatography coupled with a diode array detector (LC–DAD) according to the method described by İşleroğlu et al. (2012).

#### 2.4.9 Porosity (%) and Pore Diameter (Mm)

The porosity and pore diameter of bread samples produced under optimum conditions were determined at the Ege University Central Research Test and Analysis Laboratory Application and Research Center using a micro‐computed tomography (micro‐CT) system (Scanco Medical µCT 50, Switzerland). Three‐dimensional (3D) models were reconstructed from cross‐sectional X‐ray images of the samples. The mean pore diameter (µm) and porosity (%) values were then quantified from the reconstructed microstructural datasets.

Micro‐computed tomography analysis was performed on all bread samples obtained from the five validation baking trials conducted under the optimized air‐jet impingement and conventional baking conditions. The reported porosity values represent the mean of these validation replicates, while a representative three‐dimensional reconstruction was selected for qualitative visualization.

#### 2.4.10 Energy Consumption

Energy consumption during baking was monitored to evaluate the effect of different baking conditions. Power consumption was recorded per minute during operation at constant temperature and air velocity. Total energy consumption for each baking condition was calculated from the area under the power consumption–time curve using the trapezoidal numerical integration method implemented in Microsoft Excel (Microsoft Corp., USA) (Selçuk et al. 2026). The trapezoidal method provides an approximate estimation of total energy consumption based on discrete power measurements. Under household oven conditions, fluctuations in power draw due to thermostat cycling may introduce variability; however, this approach remains suitable for comparative evaluation of relative energy consumption between baking methods (Cruz‐Uribe and Neugebauer 2002).

#### 2.4.11 Statistical Analysis

The effects of baking conditions on bread quality parameters were evaluated using analysis of variance (ANOVA) in Design‐Expert software (Version 7.0, Stat‐Ease Inc., Minneapolis, MN, USA) for optimization of baking parameters. Data obtained from verification experiments conducted under optimum conditions were further analyzed using IBM SPSS Statistics (IBM Inc., Chicago, IL, USA) with paired‐sample t‐tests at a 95% confidence level.

## 3 Results and Discussion

**FIGURE 2:**
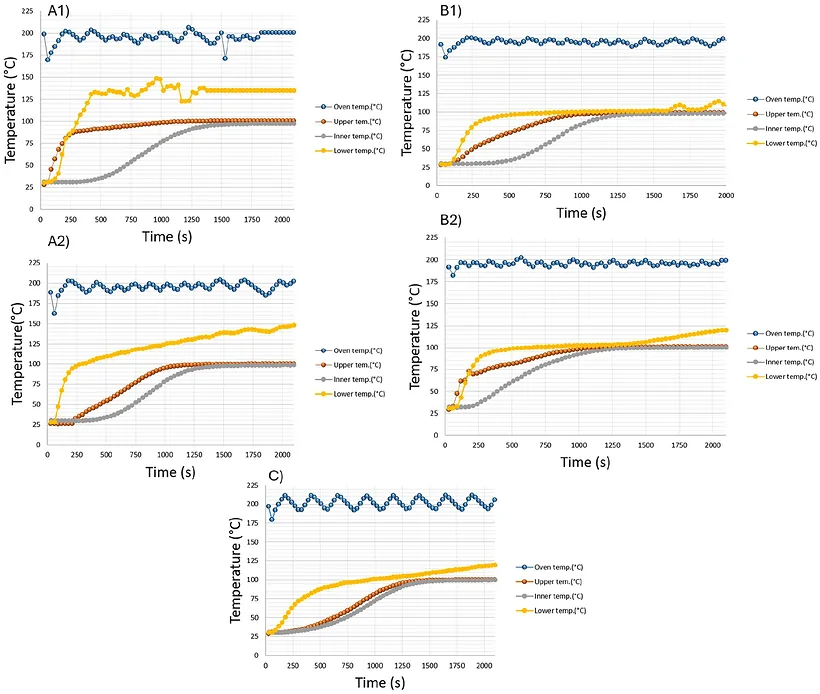
Oven temperature and upper, inner, and lower temperature profiles of bread baked under different conditions: air jet impingement cooking at 195°C for 35 min with lower tray position at 12 and 15 m/s (A1–B1) and middle tray position at 12 and 15 m/s (A2–B2), and conventional oven cooking at 200°C for 45min (C).

This section presents and discusses the effects of air jet impingement baking conditions on the physical, structural, textural, and safety‐related quality attributes of bread, with particular emphasis on the heat and mass transfer mechanisms governing these changes. The experimental results were first evaluated to determine the individual and combined effects of baking temperature, air velocity, baking time, and tray position on the selected response variables. Subsequently, the findings were interpreted using response surface methodology (RSM) and analysis of variance (ANOVA). Where relevant, the results were also compared with those obtained from conventional oven baking in order to highlight the distinctive thermal and structural behavior of the air jet impingement system. The discussion focuses on mechanistic interpretations supported by statistical analysis and relevant literature, aiming to distinguish between desirable structural development and processing‐induced quality deterioration. Figure 2 illustrates the temperature profiles recorded during baking at 195°C for 35 min, corresponding to the maximum temperature and baking time applied in the study. The figure compares temperature changes at the upper surface, geometric center (inner), and lower surface of the bread samples under different air jet impingement and conventional oven conditions.

At the same tray position, increasing the air velocity from 12 to 15 m s^−^^1^ (A1 vs. B1 and A2 vs. B2) resulted in a more rapid increase in both upper and lower surface temperatures of the bread. This behavior indicates enhanced convective heat transfer due to the higher momentum of the impinging air jets. The stronger impingement at 15 m s^−^^1^ reduces the thermal boundary layer thickness at the product surface, thereby increasing the convective heat transfer coefficient and accelerating surface heating. This effect was more pronounced at the upper surface, which was directly exposed to the impinging jets. In contrast, the internal temperature exhibited a delayed response, reflecting the dominant role of conductive heat transfer through the crumb structure.

When the effect of tray position was examined at the same air velocity (A1 vs. A2 and B1 vs. B2), bread baked at the lower tray position showed a faster increase in lower surface temperature, whereas samples placed on the middle rack exhibited a more balanced temperature increase between the upper and lower regions. This behavior can be attributed to variations in local airflow distribution and jet impingement intensity within the oven cavity, which influence the relative contributions of forced convection and conduction at different tray levels. In contrast, breads baked in the conventional oven (C) exhibited a more gradual and uniform temperature increase across the upper, inner, and lower regions. This pattern is consistent with the dominance of combined natural and forced convection together with thermal radiation and the absence of localized high‐intensity impingement jets. Overall, the air jet impingement system promoted faster surface heating and steeper temperature gradients between the crust and crumb, particularly at higher air velocities and lower tray positions. These results highlight the critical role of forced convection in governing heat transfer mechanisms during bread baking.

Table 1 presents the experimental results obtained for moisture content, height change, bulk density, and bread volume of bread samples baked under different air jet impingement conditions, including variations in baking temperature, air velocity, baking time, and tray position, together with the corresponding results obtained from the conventional oven. Table 2 summarizes the analysis of variance (ANOVA) results, indicating the significance of the main effects, interaction effects, and quadratic terms of the process variables.

**TABLE 1:** Experimental results obtained for moisture content, height change, bulk density, and bread volume of bread samples.

Temperature (^0^C)	Air velocity (m/s)	Time (min.)	Moisture content %		Height change %		Bulk density (g/cm^3^)		Bread volume (cm^3^)	
			Position							
			Middle	Lower	Middle	Lower	Middle	Lower	Middle	Lower
165	12	15	39.02 ± 0.15	37.86 ± 0.23	19.23 ± 0.17	14.41 ± 0.59	0.340 ± 0.05	0.271 ± 0.08	1290 ± 0.20	1600 ± 1.10
		25	36.76 ± 0.25	37.01 ± 0.05	9.35 ± 0.23	21.29 ± 0.05	0.240 ± 0.03	0.240 ± 0.02	1870 ± 0.47	1920 ± 0.47
		35	34.72 ± 0.37	36.17 ± 0.17	6.45 ± 0.09	14.46 ± 0.20	0.217 ± 0.01	0.131 ± 0.02	2003 ± 0.59	2002 ± 0.36
	15	15	37.63 ± 0.33	39.45 ± 0.30	15.48 ± 0.16	9.44 ± 0.36	0.235 ± 0.05	0.238 ± 0.03	1910 ± 0.96	1870 ± 0.29
		25	36.07 ± 0.80	36.54 ± 0.39	5.60 ± 0.22	5.48 ± 0.09	0.233 ± 0.05	0.231 ± 0.03	1911 ± 0.05	1890 ± 0.15
		35	32.79 ± 0.85	35.22 ± 0.15	16.34 ± 0.86	15.66 ± 0.12	0.229 ± 0.01	0.224 ± 0.08	1944 ± 0.25	1995 ± 0.36
180	12	15	38.20 ± 1.17	36.58 ± 0.96	9.36 ± 0.15	9.38 ± 0.96	0.218 ± 0.09	0.210 ± 0.04	2100 ± 0.59	1929 ± 0.15
		25	33.68 ± 0.25	33.46 ± 0.36	14.31 ± 0.45	18.60 ± 0.22	0.192 ± 0.09	0.205 ± 0.01	2290 ± 0.96	2020 ± 0.36
		35	31.45 ± 0.35	31.44 ± 0.63	14.43 ± 0.09	15.31 ± 0.05	0.187 ± 0.02	0.197 ± 0.03	2260 ± 0.12	2249 ± 0.36
	15	15	37.31 ± 1.10	37.81 ± 0.20	9.47 ± 0.17	19.38 ± 0.17	0.257 ± 0.05	0.215 ± 0.03	1610 ± 0.20	2100 ± 0.29
		25	33.84 ± 1.35	35.06 ± 0.65	5.59 ± 0.05	7.46 ± 0.63	0.260 ± 0.05	0.247 ± 0.08	1750 ± 0.59	1827 ± 0.47
		35	35.52 ± 1.17	31.69 ± 0.34	7.55 ± 0.65	15.67 ± 0.75	0.239 ± 0.02	0.204 ± 0.04	1850 ± 0.25	2190 ± 0.15
195	12	15	37.81 ± 0.72	37.63 ± 0.36	30.20 ± 0.55	33.80 ± 0.75	0.187 ± 0.05	0.155 ± 0.04	2440 ± 0.12	2800 ± 0.47
		25	31.58 ± 1.30	34.30 ± 0.36	17.23 ± 0.16	10.63 ± 0.09	0.247 ± 0.02	0.201 ± 0.08	1610 ± 0.12	2140 ± 0.15
		35	29.31 ± 1.11	31.58 ± 0.75	17.68 ± 0.65	12.37 ± 0.65	0.225 ± 0.04	0.216 ± 0.04	1950 ± 0.20	1960 ± 0.29
	15	15	38.16 ± 0.70	33.58 ± 0.63	32.17 ± 0.74	13.49 ± 0.20	0.390 ± 0.01	0.253 ± 0.15	1150 ± 0.12	1900 ± 0.47
		25	34.88 ± 0.95	35.36 ± 0.63	15.36 ± 0.69	15.35 ± 0.19	0.170 ± 0.02	0.271 ± 0.47	2490 ± 0.25	1950 ± 0.47
		35	30.55 ± 2.10	32.35 ± 0.20	11.37 ± 0.65	15.33 ± 0.17	0.240 ± 0.01	0.231 ± 0.15	1730 ± 0.20	2013 ± 0.15
	*C.O (200°C 45 min.)	30	33.37 ± 1.15		10.42 ± 0.85		0.217 ± 0.02		1950 ± 0.12	

*C.O Conventional Oven.

**TABLE 2:** ANOVA results showing the effects of processing variables on bread quality attributes.

KAYNAK	SD	Moisture content %		Height change %		Crust thickness (mm)		Bulk density (g/cm^3^)		Bread volume (cm^3^)	
		Sum of squares	p‐value	Sum of squares	p‐value	Sum of squares	p‐value	Sum of squares	p‐value	Sum of squares	p‐value
**Model**	13	363.37	< 0.0001	1054.25	0.0585	187.81	< 0.0001	0.0166	0.2777	2.99E+07	0.6383
**A**	1	77.91	< 0.0001	3.87	0.7641	15.08	0.0021	0.0015	0.1795	1.344E+05	0.0436
**B**	1	0.93	0.4814	1.29	0.8626	0.082	0.8092	0.0001	0.0735	8.22E+06	0.2032
**C**	1	257.05	< 0.0001	147.02	0.0700	136.37	< 0.0001	0.0144	0.1795	8.22E+06	0.8006
**D**	1	0.43	0.6305	1.65	0.8447	10.55	0.0090	0.0144	0.2298	1.10E+06	0.5315
A*B	1	2.66	0.2362	234.69	0.0237	2.74	0.1692	0.0072	0.0925	9.70E+05	0.1015
A*C	1	7.20	0.0549	44.42	0.3124	0.14	0.7493	0.0016	0.2383	1.204E+05	0.1242
A*D	1	2.25	0.2758	25.89	0.4393	0.15	0.7481	0.0081	0.9000	1.856E+06	0.4837
B*C	1	6.17	0.0746	20.26	0.4936	0.36	0.6156	0.0004	0.9517	4.66E+06	0.5380
B*D	1	0.015	0.9297	11.98	0.5981	0.60	0.5176	0.0000	0.7383	2.481E+06	0.7485
C*D	1	2.95	0.2124	210.01	0.0318	1.02	0.3984	0.0300	0.4844	2.601E+05	0.4908
**A^2^**	1	0.98	0.4689	46.56	0.3012	14.97	0.0022	0.0030	0.3474	1.085E+07	0.4364
**B^2^**	1	0.20	0.7417	128.47	0.0895	3.28	0.1334	0.0000		3.556E+05	
**C^2^**	1	4.61	0.1213	178.15	0.0471	2.49	0.1897	0.0011	0.5459	0.00000	0.8484
**D^2^**	0	0.000		0.000		0.000		0.0000		6.12E+06	
**Residual**	40	73.61		1697.20		55.88		0.0691		3.406E+07	
**Total**	53	436.98		2751.46		243.69		0.1856		6.404E+07	
**R^2^**		0.8315		0.3832		0.7707		0.6280		0.4683	
**R^2^_adj_**		0.7768		0.1827		0.6962		0.4339		0.1908	
C.V**		3.87		47.05		44.01		33.08		39.69	
**Press**		142.39		3270.81		100.39		149.52		4.706E+005	

A: Temperature, B: Air velocity, C: Time, D: Location, Different letters in the same column are significant at *p* < 0.05.

### 3.1 Moisture Content (%)

Moisture content is a critical quality attribute in bread, as it is closely associated with crumb softness, perceived freshness, and staling behavior. At the process level, it also reflects the intensity of coupled heat and mass transfer phenomena occurring during baking. In air jet impingement ovens, high‐velocity air jets disrupt both thermal and concentration boundary layers at the product surface, thereby enhancing convective heat transfer and accelerating moisture removal compared with conventional convection systems (Xue and Walker 2003; Sumnu et al. 2007).

Consistent with this theoretical framework, the moisture content values presented in Table 1 varied considerably under the investigated conditions, with baking temperature and baking time emerging as the primary factors governing moisture reduction. While the conventional oven required a relatively long baking period (200°C for 45 min) to achieve a moisture content of 33.37 ± 1.15%, the air jet impingement oven reached similar or lower moisture levels within shorter processing times, demonstrating its higher heat and mass transfer efficiency. Increasing the baking temperature intensified moisture evaporation by strengthening the vapor pressure gradient between the bread interior and the surrounding air, whereas longer baking times facilitated continued internal moisture migration toward the surface, in agreement with established bread‐baking mechanisms (Zhang et al. 2025). Nevertheless, the average moisture content of the bread samples obtained in all baking trials remained within the range of 30–40%, which is consistent with values typically reported for properly baked bread (Cauvain 2012).

In contrast, variations in air velocity resulted in comparatively smaller differences in the final moisture content. This observation suggests that, following rapid surface evaporation during the early stages of baking, moisture removal becomes increasingly limited by internal diffusion within the crumb structure rather than external convective transfer (Xue and Walker, 2003; Banooni et al., 2008b). The analysis of variance (ANOVA) results presented in Table 2 support these findings. Baking temperature (A) and baking time (C) were identified as highly significant factors affecting moisture content (p < 0.0001), whereas air velocity (B) and tray position (D) did not show statistically significant effects. The absence of a significant tray position effect was therefore interpreted as an indication of relatively homogeneous heat distribution within the oven cavity under the air jet impingement baking conditions.

### 3.2 Height Change (%)

Height change represents the vertical dimensional stability of bread during baking and reflects the balance between early expansion, driven by gas and vapor pressure, and subsequent shrinkage associated with crust formation and moisture loss. Accelerated surface dehydration may lead to earlier crust setting, which can restrict further expansion or promote deformation under severe thermal conditions. Consequently, a larger height change does not necessarily correspond to improved bread quality (Vanin et al. 2009; Nasser et al. 2021).

Under air‐jet impingement baking conditions, height change values exhibited a relatively wide range (approximately 5–34%), indicating that dimensional changes were strongly influenced by the processing conditions. In general, prolonged baking times tended to reduce height change values, particularly under lower‐temperature conditions, likely due to increased moisture loss and structural shrinkage during extended baking. In contrast, some higher‐temperature conditions resulted in greater height changes compared with conventional baking, which may be associated with more rapid gas expansion during the early stages of baking and accelerated crust setting. The tray position showed a comparatively limited influence on height change, further supporting the relatively uniform heating environment generated by the impingement airflow within the oven cavity. When compared with the conventional oven reference condition (200°C for 45 min, height change 10.42 ± 0.85%), impingement baking produced both lower and higher height variations depending on the severity of the applied conditions.

The non‐linear variations observed under certain baking conditions may be attributed to the simultaneous occurrence of gas expansion, moisture loss, crust formation, and localized structural collapse during baking. These findings are consistent with previous studies emphasizing the interrelated roles of moisture removal, crumb structure development, and crust formation in governing the dimensional evolution of bread during baking (Vanin et al. 2009; Chakraborty and Dash 2023). Therefore, height change should be interpreted in conjunction with volumetric parameters discussed in the following section to obtain a more comprehensive understanding of product expansion behavior.

### 3.3 Bulk Density (G/cm^3^)

Bulk density reflects the combined effects of moisture loss, gas retention, crumb expansion, and structural stabilization during baking and is an important indicator of the internal structure of bread. Lower bulk density values are generally associated with a lighter and more porous crumb structure, whereas higher values indicate reduced volumetric expansion and a denser crumb matrix (Purlis 2010; Vanin et al. 2009).

As presented in Table 1, bulk density values varied according to the applied baking conditions. In general, lower bulk density values were associated with higher bread volume and enhanced volumetric expansion, while relatively higher values indicated a more compact crumb structure. These variations may be attributed to differences in moisture removal, gas cell stability, pore development, and structural changes occurring during baking. The incorporation of whole rye flour and dark bran may also have influenced dough expansion behavior and consequently affected the bulk density of the final products. Compared with the conventional oven treatment, several air‐jet impingement baking conditions resulted in comparable or lower bulk density values at shorter processing times, indicating the potential of enhanced heat transfer to promote loaf expansion and the development of a more porous crumb structure.

The ANOVA results presented in Table 2 indicated that the evaluated processing variables did not have a statistically significant effect on bulk density (*p* > 0.05). Although differences in bulk density were observed among the baking treatments, these variations were not sufficiently large to produce significant effects within the investigated experimental range. Nevertheless, the observed tendency for lower bulk density values to coincide with higher bread volumes suggests that loaf expansion and crumb structure development were influenced by the combined effects of heat and mass transfer during baking.

### 3.4 Bread Volume (cm^3^)

Bread volume is a fundamental quality parameter reflecting the ability of dough to retain fermentation gases and steam during baking, thereby determining loaf expansion and crumb aeration. Unlike height change, which represents only one‐dimensional variation, bread volume reflects the three‐dimensional development of the loaf and is therefore considered a more reliable indicator of structural expansion.

In the present study, some variation in bread volume was observed among the air‐jet impingement baking conditions. Higher bread volumes were generally associated with lower bulk density values, indicating efficient gas retention and the formation of a well‐developed porous crumb structure. Conversely, lower bread volumes were accompanied by higher bulk density values, suggesting reduced volumetric expansion within the crumb matrix.

The observed relationship between bread volume and bulk density reflects the structural changes occurring during baking. Adequate heat transfer promotes gas cell expansion, crumb stabilization, and loaf development, resulting in increased bread volume and reduced bulk density. In contrast, insufficient expansion may limit loaf development and lead to a denser crumb structure. In the present study, all baking treatments resulted in final bread volumes that were comparable to or greater than the estimated initial dough volume, indicating successful expansion and structural development during baking.

The ANOVA results presented in Table 2 indicated that the evaluated processing variables had a limited influence on bread volume within the investigated experimental range. Although differences in bread volume were observed among treatments, the overall regression model was not statistically significant (*p* > 0.05), suggesting that the experimental factors alone could not fully explain the observed variability. This may be attributed to the complex nature of bread expansion, which is influenced by multiple interacting phenomena, including gas production and retention, dough rheology, moisture migration, and structural stabilization during baking. Similar observations have been reported in previous studies investigating bread baking processes (Purlis 2010; Vanin et al. 2009; Sumnu et al., 2007). The parallel trends observed between bread volume and bulk density further highlight the close relationship between loaf expansion and crumb structure development.

### 3.5 Hardness (N) and Crust Thickness (Mm)

**FIGURE 3:**
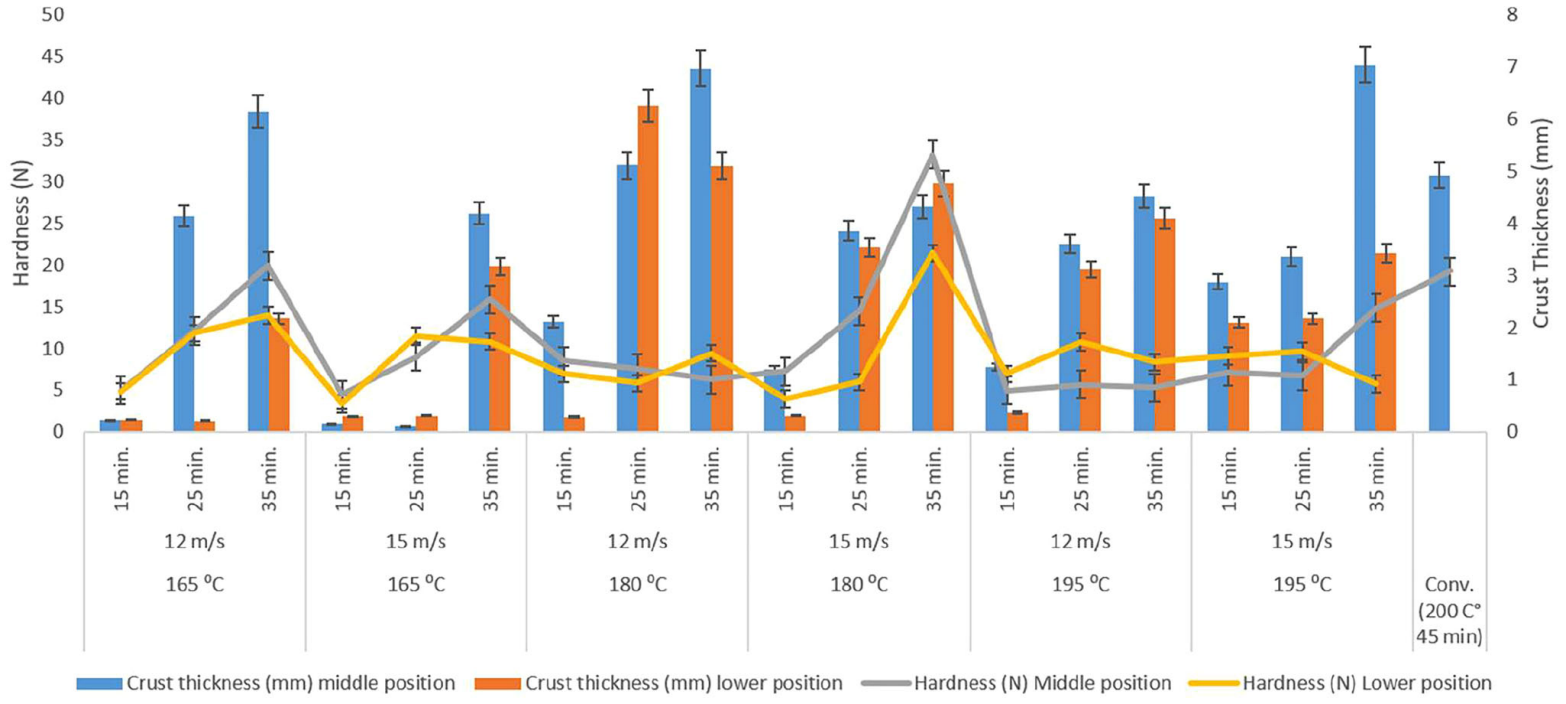
Hardness and crust thickness of bread samples baked under different air jet impingement conditions.

The hardness and crust thickness of the bread samples are presented in Figure 3. These parameters demonstrate the strong dependence of bread texture on surface dehydration and crust formation kinetics during baking. In bread systems, increased hardness is generally associated with reduced moisture content, enhanced starch gelatinization, and consolidation of the gluten–protein network, whereas crust thickness reflects the extent and rate of surface drying under thermal exposure (Cauvain 2012; Vanin et al. 2009). In air jet impingement ovens, intensified convective heat transfer generated by high‐velocity air jets accelerates surface moisture evaporation, thereby promoting earlier crust formation and modifying textural hardness compared with conventional baking systems. (Sumnu et al. 2007; Xue and Walker 2003).

As illustrated in Figure 3, increasing baking temperature and baking time generally resulted in higher hardness values and thicker crusts, which is consistent with the moisture reduction trends discussed in previous sections. Under more severe impingement conditions, rapid surface dehydration likely limited moisture migration from the crumb toward the crust, resulting in firmer textures and enhanced crust development. In contrast, milder impingement conditions preserved higher internal moisture levels, producing lower hardness values and thinner crust layers. These observations are consistent with the lower bulk density and higher bread volume obtained under similar conditions. Comparable relationships between processing severity, crust thickness, and crumb hardness have been reported for bread baked under high heat flux and forced convection environments (Purlis 2010; Sumnu et al. 2007).

Compared with the conventional oven treatment (200°C for 45 min), which produced a hardness of 19.18 ± 2.97 N and a crust thickness of about 4.92 ± 0.90 mm, air jet impingement baking resulted in a broader range of textural outcomes depending on the applied processing conditions. Several impingement treatments produced lower hardness values and thinner crust layers than the conventional reference despite significantly shorter baking times, indicating more efficient heat transfer with controlled moisture removal. Conversely, some higher temperature–time combinations generated greater hardness and thicker crusts than those obtained in the conventional oven, suggesting that excessive thermal load in impingement baking may intensify crust formation beyond desirable levels.

These findings demonstrate that, although air jet impingement technology enables faster heat transfer and improved control over crust development, careful selection of processing parameters is required to avoid excessive crust hardening and undesirable textural changes.

Table 3 presents the experimental results for hydroxymethylfurfural (HMF), a*, browning index (BI), and springiness of bread samples baked under different air jet impingement conditions, including variations in baking temperature, air velocity, baking time, and tray position, together with the corresponding results obtained using the conventional oven. Table 4 summarizes the analysis of variance (ANOVA) results, indicating the significance of the main effects, interaction effects, and quadratic terms of the process variables on the evaluated responses.

**TABLE 3:** Experimental results obtained for HMF, a*, BI and Springiness of bread samples.

Temperature(C^0^)	Air velocity (m/s)	Time (min.)	HMF (mg/kg product)		a*		BI		Springiness	
			Position							
			Middle	Lower	Middle	Lower	Middle	Lower	Middle	Lower
165	12	15	ND*	ND*	6.05 ± 1.01	5.55 ± 0.29	58.46 ± 0.57	53.97 ± 0.47	0.696 ± 0.19	0.685 ± 0.30
		25	0.12 ± 0.09	ND*	6.69 ± 2.10	6.58 ± 0.67	58.21 ± 0.19	56.77 ± 0.67	0.726 ± 0.07	0.738 ± 0.17
		35	0.47 ± 0.01	ND*	6.81 ± 1.33	6.74 ± 0.90	59.23 ± 0.33	58.78 ± 0.15	0.783 ± 0.03	0.786 ± 0.36
	15	15	ND*	ND*	6.68 ± 0.97	5.77 ± 0.55	61.04 ± 0.49	57.72 ± 0.22	0.714 ± 0.00	0.717 ± 0.52
		25	0.05 ± 0.03	0.02 ± 0.00	6.92 ± 0.69	6.75 ± 0.48	62.64 ± 0.59	60.23 ± 0.30	0.779 ± 0.01	0.747 ± 0.33
		35	0.59 ± 0.09	0.10 ± 0.04	7.20 ± 0.78	6.59 ± 0.57	63.50 ± 0.85	60.84 ± 0.08	0.77 ± 0.14	0.737 ± 0.80
180	12	15	ND*	ND*	5.95 ± 0.07	5.96 ± 0.29	63.69 ± 0.33	63.61 ± 1.65	0.713 ± 0.09	0.756 ± 0.87
		25	0.12 ± 0.04	ND*	7.61 ± 0.14	6.83 ± 0.79	66.66 ± 0.17	65.99 ± 0.97	0.81 ± 0.11	0.79 ± 0.10
		35	0.96 ± 0.24	7.06 ± 0.12	7.11 ± 0.18	7.00 ± 1.01	69.37 ± 0.32	67.37 ± 0.89	0.775 ± 0.10	0.783 ± 0.42
	15	15	0.12 ± 0.04	ND*	7.99 ± 0.15	8.16 ± 2.01	70.36 ± 0.17	69.43 ± 0.65	0.645 ± 0.22	0.731 ± 0.19
		25	0.55 ± 0.01	0.01 ± 0.00	9.01 ± 0.10	8.54 ± 1.17	73.75 ± 0.69	71.39 ± 1.15	0.823 ± 0.15	0.813 ± 0.52
		35	0.47 ± 0.25	7.08 ± 0.29	9.03 ± 0.03	8.97 ± 0.28	74.68 ± 0.09	74.15 ± 1.22	0.818 ± 0.36	0.792 ± 0.63
195	12	15	0.05 ± 0.02	ND*	8.67 ± 0.69	8.58 ± 0.40	71.06 ± 0.22	69.20 ± 0.33	0.696 ± 0.15	0.756 ± 0.85
		25	9.04 ± 0.12	0.02 ± 0.01	8.97 ± 0.65	8.78 ± 0.27	72.40 ± 0.27	72.94 ± 0.65	0.779 ± 0.36	0.821 ± 0.52
		35	6.11 ± 0.96	0.04 ± 0.02	9.09 ± 0.20	9.59 ± 0.13	73.32 ± 0.49	75.80 ± 1.17	0.805 ± 0.35	0.763 ± 0.71
	15	15	0.47 ± 0.12	ND*	9.39 ± 0.33	9.58 ± 0.17	74.61 ± 0.10	72.01 ± 1.69	0.689 ± 0.12	0.755 ± 0.08
		25	6.14 ± 0.59	3.61 ± 0.82	9.47 ± 0.85	9.20 ± 1.03	74.99 ± 0.19	72.85 ± 0.97	0.772 ± 0.36	0.77 ± 0.75
		35	12.42 ± 0.55	5.32 ± 0.29	10.29 ± 0.69	8.97 ± 0.45	77.78 ± 0.30	73.33 ± 1.10	0.821 ± 0.32	0.813 ± 0.45
	C.O** (200°C 45 min.)	30	15.03 ± 0.96		0.29 ± 0.27			90.17 ± 1.39	0.809 ± 0.34	

* ND indicates HMF concentrations below the minimum quantifiable concentration (LOQ) (<0.01 mg kg^−^^1^) of the analytical method.

** C.O Conventional l oven.

**TABLE 4:** ANOVA results showing the effects of processing variables on bread quality attributes.

KAYNAK	SD	HMF (mg/kg product)			a*		BI	Hardness (N)		Springiness	
		Sum of squares	p‐value	Sum of squares	p‐value	Sum of squares	p‐value	Sum of squares	p‐value	Sum of squares	p‐value
Model	13	308.64	< 0.0001	84.24	< 0.0001	2659.22	< 0.0001	68213.78	0.0016	0.075	< 0.0001
A	1	91.68	< 0.0001	52.97	< 0.0001	1802.85	< 0.0001	2480.82	0.2146	0.011	0.0004
B	1	4.35	0.3447	20.55	< 0.0001	621.26	< 0.0001	1790.83	0.2904	3.180E‐004	0.5074
C	1	94.02	< 0.0001	5.92	< 0.0001	119.98	< 0.0001	35051.45	< 0.0001	0.047	< 0.0001
D	1	7.25	0.2244	1.17	0.0366	40.46	0.0001	836.05	0.4684	6.827E‐004	0.3330
A*B	1	6.42	0.2523	1.29	0.0287	1.63	0.3926	6.35	0.9495	4.335E‐004	0.4395
A*C	1	51.90	0.0020	0.091	0.5499	2.13	0.3295	9092.53	0.0204	3.037E‐005	0.8373
A*D	1	43.91	0.0042	0.40	0.2146	8.90	0.0504	1199.36	0.3858	2.467E‐003	0.0699
B*C	1	3.09	0.4250	0.18	0.4062	0.55	0.6200	8034.10	0.0287	3.082E‐004	0.5141
B*D	1	0.77	0.6898	2.178E‐003	0.9262	0.45	0.6521	3779.61	0.1275	2.250E‐006	0.9554
C*D	1	0.52	0.7433	4.011E‐003	0.9000	2.36	0.3049	2488.78	0.2139	1.806E‐003	0.1188
A^2^	1	1.66	0.5580	1.03	0.0494	57.09	< 0.0001	662.57	0.5183	2.966E‐003	0.0477
B^2^	1	1.39	0.5914	0.20	0.3720	1.47	0.4167	2662.94	0.1988	2.676E‐006	0.9514
C^2^	1	1.66	0.5580	0.42	0.2055	0.096	0.8351	128.38	0.7757	8.095E‐003	0.0017
D^2^	0	0.000		0.000		0.000		0.000		0.000	
Residual	40	190.45		10.04		87.45		62401.10		0.028	
Total	53	499.09		94.27		2746.67		1.306E+005		0.10	
R^2^		0.6184		0.8935		0.9682		0.5223		0.7257	
R^2^_adj_		0.4944		0.8589		0.9578		0.3670		0.6366	
C.V**		145.21		6.72		2.27		42.02		3.51	
Press		349.27		18.76		165.85		1.120E+005		0.052	

A: Temperature, B: Air velocity, C: Time, D: Location, Different letters in the same column are significant at *p* < 0.05 * The quadratic effect of tray position was not estimable because tray position was treated as a two‐level binary factor. Therefore, Design‐Expert reports D^2^ with zero degrees of freedom and zero sum of squares.

### 3.6 Springiness

Springiness represents the elastic recovery capacity of the bread crumb after deformation and provides complementary information to hardness in the evaluation of textural quality. While hardness reflects the resistance of the crumb to compression, springiness describes the ability of the internal structure to regain its original shape. Both parameters are governed by crumb porosity, moisture distribution, and the integrity of the gluten–starch network. During baking, sufficient heat input combined with controlled moisture loss promotes structural setting while preserving elasticity; however, excessive thermal exposure or severe surface drying can increase crumb hardness at the expense of springiness.

In the present study, breads exhibiting higher hardness values generally showed lower springiness, indicating increased crumb rigidity and reduced elastic recovery under more severe baking conditions. In contrast, samples characterized by moderate hardness and higher moisture retention displayed improved springiness, reflecting a more resilient and elastic crumb structure. Statistical analysis identified baking time as a significant factor influencing springiness, whereas air velocity and tray position exhibited comparatively limited effects, suggesting that cumulative thermal load played a more dominant role than localized variations in convective heat transfer intensity.

These findings are consistent with previous studies reporting an inverse relationship between hardness and springiness in baked products, where intensified starch gelatinization and protein denaturation increase hardness while reducing elastic recovery (Scanlon and Zghal 2001; Purlis 2010). Similarly, Sumnu et al. (2007) reported that excessive moisture loss during baking leads to crumb stiffening and diminished springiness. Overall, the present results indicate that air jet impingement baking can produce a balanced textural profile by controlling thermal exposure, allowing sufficient structural development to ensure adequate hardness while maintaining elastic recovery within the crumb structure.

### 3.7 Color Development (a*) and Browning Index (BI)

Color development in bread during baking is governed by coupled heat and mass transfer processes that regulate the progression of Maillard reactions and caramelization at the product surface. As moisture evaporates from the dough surface, water activity decreases while surface temperature increases, creating favorable conditions for non‐enzymatic browning reactions responsible for changes in redness (a* value) and the Browning Index (BI). The formation of a dry crust layer further intensifies these reactions by restricting moisture diffusion and creating localized regions of high thermal severity.

In the present study, both a* and BI values generally increased with increasing baking temperature and prolonged baking time, indicating intensified surface browning under higher cumulative thermal loads. Statistical analysis showed that temperature and baking time exerted significant effects on both a* and BI, whereas air velocity and tray position exhibited limited or non‐significant main effects. This suggests that although variations in air velocity and tray position influenced surface heating rates through changes in convective heat transfer, they did not substantially alter the overall extent of browning reactions when temperature and time were controlled. The accelerated surface heating associated with air jet impingement promoted faster crust formation but did not disproportionately intensify color degradation beyond that driven by thermal severity.

The parallel increases observed in both a* and BI values reflect the progression of non‐enzymatic browning reactions and crust development during baking. Similar trends have been reported for bread and bakery products, where increasing baking temperature and time result in reduced a* values and higher BI values due to intensified non‐enzymatic browning (Purlis 2010; Vanin et al. 2009). Gökmen et al. (2008) also reported that browning intensity in baked cereal products is primarily governed by temperature–time combinations rather than airflow intensity, which supports the observations obtained in this study. Furthermore, studies on impingement and forced convection baking systems have demonstrated that enhanced heat transfer can improve process efficiency while maintaining acceptable color development, provided that excessive thermal exposure is avoided (Sumnu et al. 2007). The results indicate that air jet impingement baking enables effective control of surface color development by decoupling rapid heat transfer from excessive browning reactions. By limiting total thermal load imposed on the product while promoting uniform surface heating, this technology allows desirable crust coloration to be achieved without excessive darkening, as reflected by balanced a* and BI values.

Changes in color parameters (a* and BI) also exhibited parallel trends with HMF formation, indicating that these responses were governed by similar heat‐ and mass‐transfer‐driven mechanisms during baking. Treatments associated with lower a* values and higher BI values, reflecting intensified surface browning and crust development, also showed higher HMF levels, particularly at elevated temperatures and longer baking times. This parallel behavior suggests that the progression of non‐enzymatic browning reactions simultaneously influences both visual color attributes and the formation of HMF under conditions of reduced surface moisture and increased thermal exposure.

Statistical evaluation further confirmed that temperature and baking time were significant factors affecting a*, BI, and HMF, whereas air velocity and tray position exhibited limited or non‐significant effects. These findings indicate that although variations in convective heat transfer intensity modified surface heating dynamics, the cumulative thermal load remained the primary determinant controlling both color development and HMF formation. Similar relationships between browning intensity and HMF accumulation have been reported in bread and cereal‐based products, where progressive crust formation promotes pigment development alongside advanced Maillard reaction products (Zamora and Hidalgo 2005; Gökmen et al. 2008). Overall, the consistency observed between color parameters and HMF levels highlights that a* and BI can serve as practical indirect indicators of thermal severity during baking, linking visual quality attributes with the formation of heat‐induced chemical compounds.

### 3.8 HMF (mg/Kg Product)

Hydroxymethylfurfural (HMF) formation in bread is closely associated with the coupled heat and mass transfer processes occurring during baking. HMF originates primarily from advanced Maillard reactions and sugar dehydration pathways, which are promoted under conditions of elevated temperature and reduced moisture availability. During baking, rapid surface heating combined with moisture evaporation leads to crust formation, creating localized regions with low water activity and high temperature that are particularly favorable for HMF generation. Consequently, the extent of HMF formation depends not only on temperature but also on the cumulative thermal load and the rate of moisture removal from the bread surface.

In the present study, HMF concentration increased mainly with increasing baking temperature and prolonged baking time, indicating that total thermal load imposed on the product was the dominant factor governing HMF formation. Statistical analysis confirmed that temperature and baking time exerted significant effects on HMF content, whereas air velocity and tray position did not show significant main effects. This suggests that although variations in air velocity and tray position modified local convective heat transfer rates and surface heating dynamics, they did not substantially increase the overall thermal severity required to intensify HMF‐forming reactions. The enhanced convective heat transfer associated with air jet impingement primarily accelerated heating and drying rates without extending the residence time of surface layers at critical temperatures, thereby limiting excessive HMF accumulation.

These observations are consistent with previous studies reporting that HMF formation in bread and cereal‐based products is predominantly governed by temperature–time combinations rather than airflow intensity alone (Zamora and Hidalgo 2005; Gökmen et al. 2008). Capuano and Fogliano (2011) also emphasized that prolonged exposure to high temperatures under low‐moisture conditions is necessary for significant HMF accumulation, whereas shorter, high‐intensity heating treatments tend to restrict its formation. Similar conclusions have been reported for high‐intensity convective and impingement‐based baking systems, where improved heat transfer efficiency shortens processing time and thereby mitigates the formation of heat‐induced contaminants despite higher local heat fluxes (Sumnu et al. 2007). Overall, the present results demonstrate that air jet impingement baking can effectively control HMF formation by enabling rapid heat transfer while avoiding excessive thermal exposure. This highlights the critical role of heat–mass transfer interactions in limiting undesirable chemical reactions during bread baking.

### 3.9 Energy Consumption (Wh)

**FIGURE 4:**
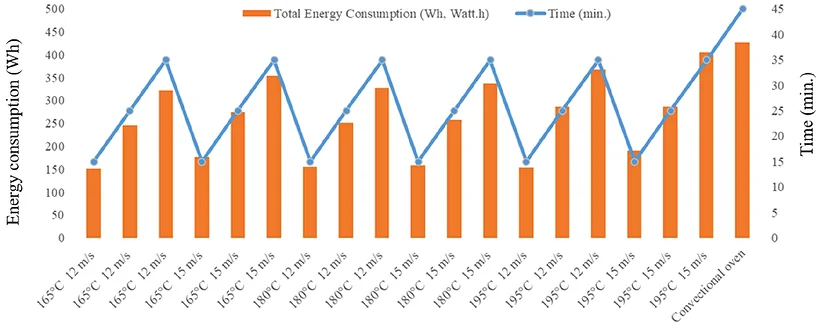
Total energy consumption (Wh) and corresponding baking time (min) of bread samples baked under different air jet impingement conditions at varying temperatures and air velocities, in comparison with conventional oven baking.

Figure 4 presents the total energy consumption and corresponding baking times for the different air jet impingement baking conditions, together with the results obtained using the conventional oven. As expected, total energy consumption increased with longer baking times and higher processing temperatures, reflecting the cumulative nature of energy use during thermal processing. Across all air jet impingement treatments, a strong positive relationship between baking time and total energy consumption was observed, indicating that processing time was the dominant factor influencing overall energy demand.

At a given temperature, increasing the air velocity from 12 to 15 m/s^−^^1^ generally resulted in a slightly higher instantaneous power demand but did not proportionally increase the total energy consumption, due to the shorter baking times enabled by enhanced convective heat transfer. This behavior highlights the efficiency of air jet impingement systems, in which higher heat transfer coefficients accelerate product heating and reduce the required processing duration, thereby limiting cumulative energy consumption despite the increased airflow intensity. In contrast, lower air velocities required longer baking times to achieve comparable product conditions, resulting in higher overall energy consumption.

Temperature also exerted a significant influence on energy use. Although higher temperatures increased instantaneous energy input, they frequently reduced the required baking time under air jet impingement conditions. As a result, total energy consumption values remained lower than or comparable to those obtained at lower temperatures combined with longer baking durations. These observations emphasize that optimizing temperature–time combinations is critical for minimizing energy consumption in high‐intensity baking systems.

The conventional oven exhibited the highest total energy consumption, primarily due to its substantially longer baking time. Despite operating at a comparable or slightly higher temperature, the lower heat transfer efficiency characteristic of conventional ovens prolonged the baking process and increased the cumulative energy demand. This contrast highlights a key advantage of air jet impingement technology, which enhances heat transfer efficiency and thereby reduces processing time without increasing overall energy consumption.

Overall, the results demonstrate that air jet impingement baking provides a significant energy efficiency advantage by shortening processing times through intensified convective heat transfer. When appropriately optimized, this technology can substantially reduce total energy consumption while maintaining effective baking performance, supporting its potential as a more energy‐efficient and sustainable alternative to conventional oven systems.

### 3.10 Determination of Optimum Baking Conditions

Optimization of bread baking conditions in the air‐jet impingement oven was performed using Design‐Expert software (Version 13.0, Stat‐Ease Inc.) based on experimental data obtained under different processing conditions. The independent variables considered in the optimization were oven temperature (165, 180, and 195°C), air velocity (12 and 15 m/s^−^^1^), baking time (15, 25, and 35 min), and tray position (middle and lower racks). The response variables selected for the optimization were crust thickness (mm), bread volume (cm^3^), and hardness (N), as these parameters directly represent product quality and consumer acceptance. During optimization, bread volume was maximized, whereas hardness and crust thickness were minimized to obtain breads with desirable structural properties. The experimental data for each response variable were fitted to a quadratic model (Equation 1) using Design‐Expert software, and the significance of the model terms was evaluated using analysis of variance (ANOVA). The adequacy of the fitted quadratic models was assessed based on the coefficient of determination (*R* ^2^), adjusted coefficient of determination (*R* ^2^adj), coefficient of variation (C.V.%), and other error‐related statistics, as reported in Tables 2 and 4.

The crust thickness model exhibited satisfactory predictive performance, with R^2^ and adjusted R^2^ values of 0.7707 and 0.6992, respectively. In contrast, lower *R* ^2^ and adjusted *R* ^2^ values were obtained for bread volume (0.4683 and 0.1908) and hardness (0.5223 and 0.4672), indicating substantial experimental variability and limited predictive capability of the developed models for these responses. In particular, bread volume is influenced by complex interactions among dough structure, gas retention, moisture migration, dough rheology, and expansion behavior during baking, which may contribute to the observed variability and reduce the predictive performance of empirical regression models. Similar limitations are commonly reported for quality attributes that are strongly affected by biological and structural variability, especially in fiber‐rich bakery formulations.

The coefficient of variation (C.V.%) is commonly used as an indicator of the degree to which experimental data are clustered around the mean value (Lazić 2004). The calculated C.V. values were 44.01% for crust thickness, 39.69% for bread volume, and 42.02% for hardness. These relatively high values may be attributed to the inherent structural heterogeneity and measurement variability associated with bread systems, where variations in crumb structure, moisture distribution, and crust formation may introduce experimental fluctuations.

**FIGURE 5:**
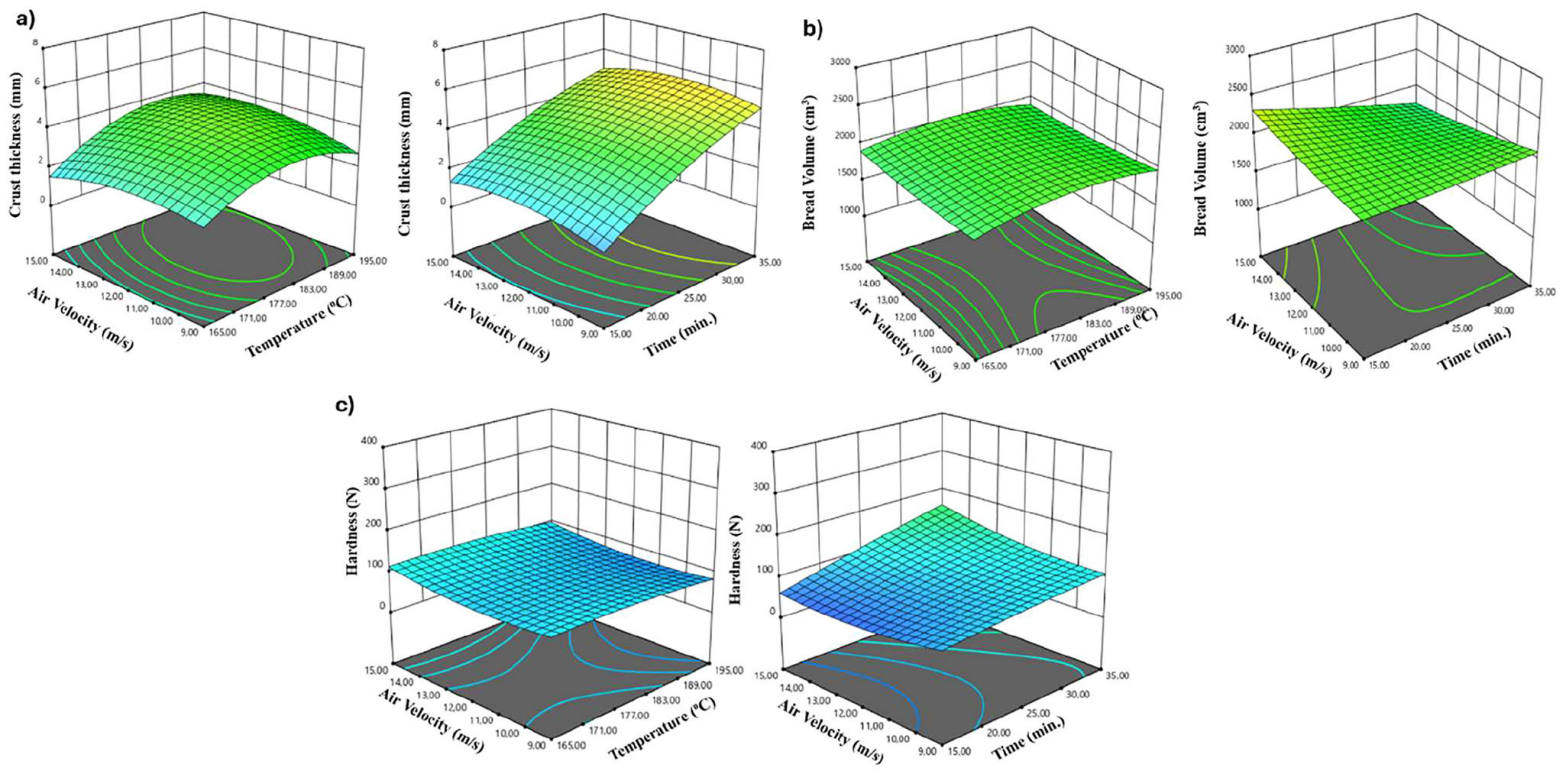
Response surface and contour plots for a) crust thickness, b) bread volume and c) hardness as a function of air jet impingement baking parameters at the position of middle tray.

The predictive capability of the regression models was further evaluated using the Prediction Error Sum of Squares (PRESS) statistic. Lower PRESS values indicate higher predictive accuracy of the model (Myers and Montgomery 1995). Accordingly, the regression model exhibited stronger predictive performance for crust thickness, whereas the models developed for bread volume and hardness showed greater prediction uncertainty due to the higher variability associated with these responses. Therefore, these models should primarily be interpreted for identifying general response trends rather than for precise prediction. To visualize the effects of the baking variables on the selected responses, response surface and contour plots were generated using the developed quadratic models. These plots are presented in Figure 5.

As shown in Figure 5, crust thickness increased with increasing temperature and air velocity up to a certain point, after which a decreasing trend was observed. This behavior may be attributed to the operating principle of air jet impingement ovens, in which high‐velocity jets deliver heat directly to the product surface, accelerating crust formation, particularly during the early stages of baking. Consistent with this interpretation, longer baking times further increased crust thickness, indicating enhanced crust development under prolonged thermal exposure.

Bread volume showed some variation with temperature, air velocity, and baking time. However, the response surfaces indicated relatively small changes within the investigated experimental range, suggesting that these processing variables had a limited influence on bread volume. This observation is consistent with the ANOVA results, which indicated that the overall bread volume model was not statistically significant (*p* > 0.05). Although hardness was not significantly influenced by temperature, it showed a slight increase up to intermediate temperature levels, followed by a less pronounced decreasing tendency. Unlike crust thickness, the effect of air velocity on hardness did not follow a similar pattern. Instead, hardness slightly decreased with increasing air velocity before increasing again. This behavior may be explained by the fact that hardness measurements were performed on bread slices containing both crust and crumb, rather than on the crust alone. Under certain conditions, enhanced crumb softness may partially offset the hardening effect associated with crust development, resulting in reduced overall hardness values.

The effect of baking time on hardness was found to be approximately linear, indicating progressive structural firming with prolonged thermal exposure. The optimal baking conditions were determined using the desirability function approach, with the objective of maintaining crust thickness within an acceptable range while minimizing hardness and maximizing bread volume. Based on the optimization analysis, the optimum baking parameters were determined as 195°C oven temperature, 13.32 m s^−^^1^ air velocity, 35 min baking time, and lower tray position, corresponding to a desirability value of 0.793. To validate the model predictions, five additional baking trials were conducted under the identified optimum conditions. The predicted and experimental values for each response variable were compared using a one‐sample t‐test performed in IBM SPSS Statistics Base 22.0. The results of this validation analysis are presented in Table 5, confirming the adequacy of the developed optimization model.

**TABLE 5:** Results of statistical analysis for verification of optimization.

Responses	Predicted value	Experimental value^a^	SE^b^	Difference	% Error^c^	*p*‐value
**Crust thickness (mm)**	4.125	4.348 ± 0.271	0.121	−0.187	4.533	0.198
**Bread volume (cm^3^)**	1863.87	1865.56 ± 10.967	4.905	4.905	4.905	4.905
**Hardness (N)**	8.972	8.710 ± 0.332	0.148	0.260	2.985	0.154

^a^ Experimental values were expressed with standard deviation, ^b^Mean standard error.

^c^ % Error = (│yexp—ypre│/ yexp) * 100;  yexp: experimental value, ypre: predicted value.

Based on the results presented in Table 5, the average experimental values obtained under the optimum baking conditions did not differ significantly from the model‐predicted values (*p* > 0.05). This agreement confirms the adequacy, reliability, and predictive capability of the developed optimization model.

### 3.11 Evaluation of Acrylamide Content and Porosity of Bread Produced under Optimum Baking Conditions

Due to their complex structure and relatively low thermal conductivity, bread and other dough‐based products often experience non‐uniform heat distribution during conventional baking. In such systems, thermal reactions associated with baking tend to occur earlier and more intensely at the outer layers than within the interior, which can hinder homogeneous baking. When the baking process is prolonged to ensure adequate internal baking, excessive surface drying and intensified Maillard reactions may occur, potentially leading to the formation of process contaminants such as acrylamide in cereal‐based foods (İşleroğlu et al., 2012). According to the scientific opinion report on acrylamide in foods published by the European Food Safety Authority (EFSA 2015), the indicative acrylamide value for wheat bread is 80 µg kg^−^^1^.

In addition to chemical safety aspects, the porosity of bread crumb is a critical structural parameter in high‐volume bakery products. Porosity reflects the amount, size, and spatial distribution of gas cells formed within the crumb matrix and plays a direct role in determining product volume, crumb structure, textural characteristics, and consumer acceptance (Scanlon and Zghal 2001).

**FIGURE 6:**
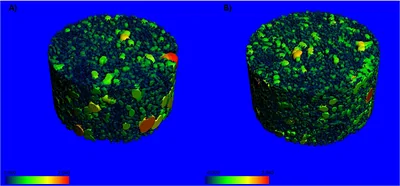
Crumb pore structure images of breads baked in air jet impingement under the optimum conditions (A) and conventional oven (B).

In the present study, acrylamide and porosity analyses were conducted to evaluate how baking processes performed in conventional and air jet impingement ovens influence not only the quality characteristics of bread but also the formation of acrylamide, a key process contaminant in terms of food safety, as well as the porous morphology of the crumb structure. Accordingly, the objective was to comparatively assess how differences in heat and mass transfer characteristics between the two baking technologies affect both the potential formation of acrylamide and the development and distribution of gas cells within the bread crumb. The acrylamide content and porosity characteristics of breads produced under the optimum processing conditions in the air jet impingement oven and the conventional oven are summarized in Table 6. In addition, the corresponding crumb pore structure images are presented in Figure 6, providing a visual comparison of the internal crumb morphology obtained under each baking system.

**TABLE 6:** Comparison of acrylamide content (mg/kg), porosity (%), and pore diameter (mm) of breads produced under the optimum air jet impingement baking conditions and conventional oven baking.

Baking conditions	Porosity (%)	Pore diameter (mm)	Acrylamide (mg kg^−1^)
Air jet impingement oven (195^0^C, 13.32 m/s, 35 min., lower position)	64 ± 1.96	0.589 ± 0.01	ND*
Conventional oven (200°C 45 min.)	55.93 ± 1.22	0.412 ± 0.05	ND*

ND indicates acrylamide concentrations below the LOQ (<0.1 mg kg^−^^1^) of the analytical method.

As shown in Table 6 and Figure 6, the bread sample baked under the optimum air jet impingement conditions exhibited a more pronounced and porous crumb structure compared with the sample baked in the conventional oven. The porosity value of bread produced in the air jet impingement oven reached 64%, whereas the porosity of the conventionally baked sample was 55.93%. In a study conducted by Chen et al. (2021), porosity values of bread baked in an industrial oven were reported as 53.7%, which is consistent with the results obtained for the conventional baking condition in the present study. In contrast, Van Dyck et al. (2014) reported porosity values of approximately 73% for bread samples, which are higher than those observed in this study. These differences can be attributed to variations in proofing conditions, formulation, and processing parameters, which are known to significantly influence crumb structure. The average pore diameter was determined as 0.589 mm for the bread baked in the air jet impingement oven and 0.412 mm for the sample baked in the conventional oven. Under air jet impingement conditions, gas cells appeared larger and more uniformly distributed, which is consistent with the formation of a more open and higher‐volume crumb structure. In contrast, the conventionally baked sample exhibited smaller pores and a denser crumb morphology, resulting in a more compact internal structure. This structural difference can be attributed to the enhanced heat and mass transfer conditions provided by the air jet impingement system, particularly at higher air velocities. The intensified convective heat transfer promotes more rapid gas expansion and improved stabilization of the crumb structure during baking, thereby contributing to the development of a more porous crumb.

In addition, acrylamide was not detected under either baking condition, indicating that the applied processing parameters remained within a safe range with respect to acrylamide formation. This finding suggests that the optimized air jet impingement baking process can improve crumb structure and porosity without increasing the risk of heat‐induced chemical contaminants.

## 4 Conclusion

This study systematically evaluated the effects of air jet impingement baking on the physical, structural, textural, color, and safety‐related quality attributes of bread, with a focus on the interplay between heat and mass transfer mechanisms. Compared with conventional oven baking, the air jet impingement oven enabled faster surface heating, steeper temperature gradients, and more efficient moisture removal, allowing comparable or improved bread quality to be achieved within shorter baking times and with lower energy consumption.

Under optimized conditions (195°C, 13.32  m/s air velocity, 35 min, lower tray position), bread exhibited higher porosity (64%), larger and more uniform pore distribution, and enhanced crumb elasticity, while maintaining acceptable crust thickness and hardness, demonstrating the technology's capability to support structurally uniform and high‐volume loaves. Color and hydroxymethylfurfural (HMF) analyses indicated that the intensified heat transfer accelerated surface browning without excessive formation of undesirable Maillard reaction products. Similarly, acrylamide was not detected in any sample, confirming that the optimized air jet impingement baking process remained within safe processing limits.

Response surface methodology and statistical analyses confirmed the predictive reliability of the developed models, highlighting the dominant roles of temperature and baking time in controlling bread quality, while air velocity and tray position played secondary roles. Overall, the findings demonstrate that air jet impingement technology offers a promising alternative to conventional baking, providing improved process efficiency, structural quality, and energy sustainability while maintaining food safety. The results give useful insights for both industrial bakery applications and further research on heat–mass transfer–driven optimization of baked products.

## Author Contributions

**Özgül Altay**: conceptualization, investigation, methodology, writing – original draft, writing – review and editing, and formal analysis. **Özge Filiz**: conceptualization, writing – original draft, writing – review and editing, investigation, methodology, and formal analysis. **Gönül Çavuşoğlu**: conceptualization, investigation, methodology, software, resources, writing – review and editing. **Safiye Nur Dirim**: conceptualization, investigation, validation, writing – review and editing, supervision, and data curation. **Utku Şentürk**: conceptualization, investigation, methodology, validation, software, writing – review and editing. **Figen Kaymak‐ertekin**: conceptualization, project administration, resources, supervision, data curation, and validation.

## Conflicts of Interest

The authors declare no conflicts of interest.

## Data Availability

Data supporting the findings of this study are available upon request from the corresponding author.
